# Revisiting the Therapeutic Effects of Essential Oils on the Oral Microbiome

**DOI:** 10.3390/pharmacy11010033

**Published:** 2023-02-10

**Authors:** Casandra-Maria Radu, Carmen Corina Radu, Sergiu-Alin Bochiș, Emil Marian Arbănași, Alexandra Ioana Lucan, Viorela Romina Murvai, Dana Carmen Zaha

**Affiliations:** 1 Doctoral School of Biological and Biomedical Sciences, University of Oradea, 1 University Street, 410087 Oradea, Romania; 2 Department of Forensic Medicine, George Emil Palade University of Medicine, Pharmacy, Science, and Technology of Targu Mures, 38 Gheorghe Marinescu Street, 540139 Targu Mures, Romania; 3 Doctoral School of Medicine and Pharmacy, George Emil Palade University of Medicine, Pharmacy, Sciences and Technology of Targu Mures, 540142 Targu Mures, Romania; 4 Clinic of Vascular Surgery, Mureș County Emergency Hospital, 540136 Targu Mures, Romania; 5 Department of Vascular Surgery, George Emil Palade University of Medicine, Pharmacy, Science, and Technology of Targu Mures, 38 Gheorghe Marinescu Street, 540139 Targu Mures, Romania; 6 Faculty of Medicine and Pharmacy, Department of Preclinical Disciplines, University of Oradea, 1 December Sq, 410028 Oradea, Romania

**Keywords:** essential oils, therapeutic effect, oral microbiome, phytopharmaceuticals, chemical composition, antibiotic resistance, dental diseases

## Abstract

The extensive use of antibiotics has resulted in the development of drug-resistant bacteria, leading to a decline in the efficacy of traditional antibiotic treatments. Essential oils (EOs) are phytopharmaceuticals, or plant-derived compounds, that possess beneficial properties such as anti-inflammatory, antibacterial, antimicrobial, antiviral, bacteriostatic, and bactericidal effects. In this review, we present scientific findings on the activity of EOs as an alternative therapy for common oral diseases. This narrative review provides a deeper understanding of the medicinal properties of EOs and their application in dentistry. It not only evaluates the effectiveness of these oils as antibacterial agents against common oral bacteria but also covers general information such as composition, methods of extraction, and potential toxicity. Further nonclinical and clinical studies must be conducted to determine their potential use and safety for treating oral diseases.

## 1. Introduction

Microbes were discovered in the early 18th century and are prevalent in our environment, affecting every aspect of human life. The oral cavity is home to various microorganisms and habitats that play a crucial role in overall human health. Imbalances in the microbial flora can lead to oral diseases such as dental cavities, periodontitis, gingivitis, oral mucosa diseases, and systemic diseases [1]. Many attempts have been made to develop the ideal antimicrobial agent due to the emergence of antibiotic-resistant bacteria [2]. EOs have been studied for many years as potential antimicrobial agents and are used in various medical fields, including dentistry. In many countries, they are still used as traditional medicine. The earliest known use of EOs is believed to be in Ancient Egypt in 3500 B.C., where they were used in cosmetics, religious ceremonies, and medicinal purposes in various forms such as ointments, inhalations, powders, pills, and maceration extracts [3]. French chemist Rene-Maurice Gattefosse experimented with EOs for wound healing during World War I [4]. India and China also began using herbs as a medicine around the same time as Ancient Egypt, and currently, there is an increasing demand and interest in “natural medicine” due to concerns about synthetic drugs, fertilizers, and pesticides [5]. However, the use of aromatherapy for emotional and mental well-being gained popularity in the 1980s when research on mind–body healing and psychoneuroimmunology increased interest in the potential benefits of aromatherapy. It is commonly believed that certain scents can affect a person’s emotional state [6,7].

Approximately 3000 EOs are known to be used, and their use is increasingly studied now due to the need for alternative therapies for oral microbiome pathologies [8]. According to the World Health Organization, about 80% of the population uses herbal medicine, and its industrialization has highly increased [9]. EOs are effective as antioxidants, mostly because of their activity in food preservation [10], and they are known to possess anti-carcinogenic, antimicrobial, and anti-inflammatory properties due to over 200 constituents [11,12]. EOs are a mixture of volatile constituents produced by aromatic plants, serving as a protective mechanism against microorganisms [13]. Clove oil, also known as Eugenol in dentistry, is an aromatic oil extracted from cloves that have been proven to be very useful in root canal treatments in the past decade. However, many more EOs are now being studied for their therapeutic use, such as *Tea tree oil*, *Thyme oil*, *Cinnamon oil*, *Citrus oil*, *Bergamot oil*, *Lavender oil*, and *Peppermint oil*. In dentistry, the most common pathologies are bacterial and fungal, with pathogens such as *Streptococcus mutans* (*S. mutans*), *Streptococcus salivarius* (*S. salivarius*), *Streptococcus sanguis* (*S. sanguis*), *Streptococcus sobrinus* (*S. sobrinus*), *Porphyromonas gingivalis* (*P. gingivalis*), *Prevotella intermedia* (*P. intermedia*), *Enterococcus faecalis* (*E. faecalis*), *Candida albicans* (*C. albicans*), and *Actinobacillus actinomycetemcomitans* (*A. actinomycetemcomitans*) often modifying the oral microbiome and resisting other known therapies [2,14]. Increased bacterial resistance, the high costs of therapeutic procedures, and the many adverse effects have led to further research on traditional medicines obtained from plant sources [15,16]. Despite the widespread use of commercial drugs as trusted therapies, many people still use natural products for primary healthcare [17].

The oral cavity is a habitat for many microorganisms that form a complex structure, the biofilm, that adheres to teeth and oral epithelium. Oral diseases occur when there is an imbalance between the oral ecosystem and the biofilm; thus, the absence of microorganisms is preferred to maintain oral health [18]. As a result, natural agents have become necessary, making EOs great alternatives to antibiotics and other used therapies, such as photoactivation and lasers [19]. This narrative review will describe and discuss more information about EOs.

This current paper aims to collect literature reviews about the therapeutic effects of EOs on the oral microbiome concerning its diverse field of conditions, such as dental cavities, candidiasis, gingivitis, periodontitis, and oral cancer, and to highlight their benefits to combat antibiotic resistance. Additionally, this paper will point out which EOs can be used in dental treatments as an alternative to antibiotics and how dentists can benefit from them.

## 2. Main Body

### 2.1. Materials and Methods

This paper aims to answer the following question: “*What is the therapeutic effect of EOs on the oral microbiome based on evidence gathered from existing articles*?”. According to a set strategy, a narrative review was conducted using the database platforms PubMed, PC, ScienceDirect, Scopus, NCCIH, and Wiley Online Library utilizing the following key terms: therapeutic effect, essential oils, oral microbiome, and new therapies. The search was performed from January 2022 until June 2022. An initial literature review resulted in 1560 articles, 136 remaining after the screening. Articles were eligible only if they were written in English and published between 2010 and 2023. Further unpublished work was not necessary to be found.

### 2.2. Generalities and Extraction Methods

The positive health effects of EOs have been known since ancient times [20]. The earliest recorded mention of the methods used to produce EOs is believed to be that of Ibn al-Baitar (1188–1248) [21]. EOs usually come from seeds, stems, leaves, flowers, petals, fruits, woods, resins, roots, rhizomes, and grasses [22]. The active part of the plant which contains the functional particles is obtained during the extraction, together with the residual part. The raw extracts are alkaloids, phenolic compounds, flavonoids, glycosides, and terpenoids [23,24].

The methods are specific to their hydrophobic and volatile nature, and they are named “plant extracts” preceded by the name of the technique that is being used [25,26]. As shown in Table 1, the extraction methods are advanced and conventional; the advanced methods are preferred due to less extraction time, low energy consumption, low solvent uses, and less carbon dioxide emission [11]. The method known as hydrodistillation consists of microwave-assisted hydrodistillation (MAH) and Clevenger hydrodistillation (CH); from these two, MAH is nine times faster compared with CH. In addition, it obtains the exact yield of EOs in twenty minutes [27]. EOs can also be isolated using hydrolysis, crushing, extraction, and fermentation [28].

### 2.3. Composition

EOs, also known as “volatile oils,” are produced by aromatic plants as secondary metabolites and are characterized by their strong smell [36]. The chemical composition varies and depends on geographical location, botanical origin, genetics, bacterial endophytes, and extraction techniques [21]. They are synthesized from plants, especially from their leaves, fruits, resins, seeds, woods, barks, and berries, and they are known as “essentials” because they trap the essence of the plant, its taste, and its odor [37]. They have attracted the interest of research groups because they can be applied to the development of new solutions used for the improvement of oral hygiene [38]. EOs are complex substances that include hundreds of components [10] but are characterized by two or three significant compounds [39].

The main composition is made of hydrocarbon terpenes and terpenoids [25,40], and other common compounds are alcohols, acids, esters, epoxides, aldehydes, ketones, amines, sulfides, oxides, fatty acids, other sulfur derivates; the most critical ones for their activities are terpineol, thujanol, myrcenol, neral, thujone, camphor, carvone [20,41]. The majority of terpenoids consist of monoterpenes and sesquiterpenes, and the other group is oxygenated derivatives of hydrocarbon terpenes [25]. Due to their potential therapeutic benefits against various illnesses, monoterpenes have been the subject of extensive research [42]. EOs have been proven to be a valuable source of antitumor agents. In addition, their effectiveness in both mechanisms of action and clinical use in cancer treatment has been demonstrated [43]. The bactericide or bacteriostatic effects are due to terpenes and terpenoids, aromatic, and aliphatic constituents [44], and the antimicrobial activity is related to their composition, configuration, amount, and possible interactions [45]. The antimicrobial activities might also be due to their major phenolic or alcohol monoterpenes components [46], but Table 2 explains that in more detail.

### 2.4. Applications

Oral health refers to the health of the teeth, gums, tongue, cheeks, and the entire oro-facial system that provides the human physiological functions. The most common dental diseases are dental cavities, periodontitis, gingivitis, and oral cancer, and EOs seem to have a beneficial role in each one of them, as seen in Table 3. Even though the research area is quite large, further clinical trials must be performed before using these EOs as therapeutic agents [59].

Dental cavities are one of the leading global public health problems; the first step of dental cavities and periodontitis is the accumulation of microbial plaque on dental surfaces. Next, the bacteria produce acids which progress further destruction of the teeth. There are about twenty-five species of Streptococci in the oral cavity, from which *S. mutans* and *S. sobrinus* have a direct association with tooth decay [60].

**Table 3:** Dental diseases and EOs uses.

Dental Disease	EOs	Therapeutic Effect	Reference
Dental cavities	*Clove oil* *Sesame oil* *Cinnamon oil* *Sumac oil* *Citrus oil*	antibacterial antimicrobial antifungal anticariogenic antiadhesion properties	[37,59,61,62]
Periodontitis	*Clove oil* *Lavender oil* *Lemongrass oil* *Eucalyptus oil*	anti-inflammatory antibiofilm growth effect	[15,28,37,63]
Dental pain	*Lavender oil* *Clove oil*	anxiolytic analgesic-like effect anti-inflammatory	[37,64,65,66]
Oral cancer	*Clove oil* *Cinnamon oil*	anti-inflammatory antimutagenic cytotoxic immunomodulatory	[67,68,69,70]

### 2.5. Therapeutic Properties

**Figure 1:**
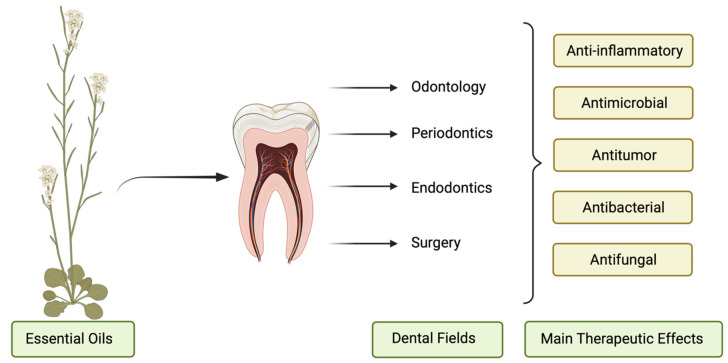
The therapeutic effects of EOs in diverse fields of dentistry.

The applications of EOs depend on the plant source and are very diverse. They are also used in cosmetics and in the food and pharmaceutical industries. In addition, they have immunomodulatory effects by increasing the number of circulating lymphocytes [71]. A certain number of EOs have been reported to be antibacterial, antifungal, and anti-inflammatory agents against oral pathogens, and other therapeutic effects are shown in Figure 1 [72]. Additionally, they can alleviate anxiety, depression, and nausea [73,74,75].

EOs are found to be most efficient against S. mutans, followed by *S. sobrinus*, *salivarius*, *sanguis*, and *Lactobacillus acidophilus (L. acidophilus)* [13]. EOs have also been tested against *C. albicans*, but only a few studies have been conducted on their activity [76,77]. *Oregano oil* was found to prevent the adhesion and formation of *C. albicans* biofilm. It also reduced biofilm formation on surfaces previously treated with the oil [72].

The primary antimicrobial mechanisms of EOs are associated with increased cell membrane permeability; this results in the extravasation of ions and cellular contents and cell lysis [78]. EOs damage cells differently by changing the structure and function of the membrane or by interfering with the cell metabolism and causing its death [72]. They can also interfere with protein synthesis or cell division by stimulating the production of reactive oxygen species [78].

Studies have shown that EOs also have antiviral effects on several viruses: Coxsackie, HAdV, HCMV, HIV, HSV (1 and 2), HINI, SARS-CoV, VSV, and YF, but further studies have to comply [71,73,79].

### 2.6. Uses of EOs as Products in Dentistry

**Figure 2:**
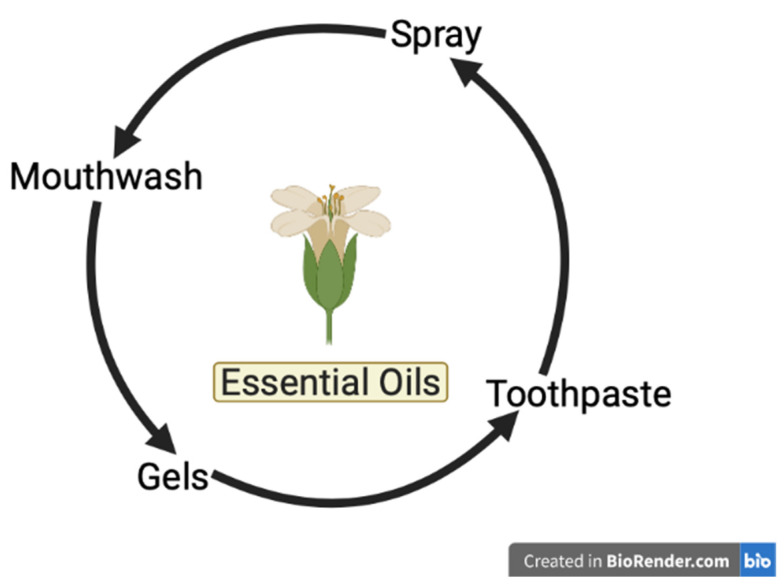
EOs found in dental products.

EOs are recognized as safe, and they stimulated searchers as a natural treatment of dental diseases [72]. However, despite the research progress that has been performed until now, studies regarding EOs’ approaching potential application in dentistry are still not discussed enough [13]. EOs are very useful in dentistry in the following fields: endodontics, periodontics, surgery, and oral prevention [80], and can be found in several dental products, as shown in Figure 2. They are known to be useful as oral hygiene adjuncts, anxiolytics, wound dressing, dental implants, and preservatives.

#### 2.6.1. Oral Hygiene Adjuncts

EOs have been used since the 19th century in dentistry as a mouthwash for the prevention of dental diseases. Bacterial counts in saliva dropped 10–20% after rinsing and remained efficient for 7 to 12 h [81]. A randomized clinical trial found that the daily use of an EO-based mouthwash can significantly reduce plaque, gingivitis, and periodontitis more than 0.05% cetyl pyridinium chloride-containing mouth rinse [82]. A short daily application of EO mouthwash rinses is not harmful and has no irritation potential [83], but some clinical trials showed that they possess different degrees of cytotoxicity [84]. EOs seem to have a plaque-inhibitory effect, so the soft tissues would gain supplementary protection against bacterial attack [85]. Even if chlorhexidine (CHX) tends to be the first choice for plaque control and the management of gingivitis and periodontitis, the most reliable alternative is EOs; CHX provides tooth discoloration, the desquamation of oral mucosa, taste disturbances, and supragingival calculus deposition so that EOs could be preferred [86,87]. EOs in mouthwashes kill viruses by disrupting the phospholipid bilayer, altering the viral envelope, and spiking proteins to prevent the virus from attaching to host cells. The main side effects of using EO mouthwashes are a burning sensation and temporary enanthema [79]. *Lavender oil* also has solid antiseptic properties against *Staphylococcus aureus (S. aureus)* and *Enterococcus coli (E. coli)* [51]. However, for *Candida albicans (C. albicans)*, more studies need to be conducted [8,88]. It is used in mouth, throat, and upper respiratory tract infections by showing substantial antibacterial effects. *Thyme oil* showed antiviral properties against the Herpes simplex virus and had bacteriostatic and antimicrobial effects [28,48]. Citrus fruits such as sweet orange, bitter orange, lemon, lime, grapefruit, bergamot, yuzu, and kumquat are found to be effective as medicinal agents in mouthwashes, too; they have the following properties: anti-tumor, antibacterial, antifungal, larvicidal, antioxidant, anti-carcinogenic, and anti-inflammatory effects, but the data based on oral pathology are not shown yet [89,90]. Other studies concluded that even if the natural-based mouth rinses have plaque-inhibitory potential, the gold standard remains CHX-based mouthwashes [87,91,92].

#### 2.6.2. Anxiolytics

Aromatherapy, a form of complementary therapy, is widely used in many countries and involves using EOs through inhalation, skin absorption, or ingestion for preventive and active medical care. In recent years, it has alleviated insomnia, depression, anxiety, and cognitive disorders. In addition, accumulating evidence over the past decade has demonstrated that EOs have measurable pharmacological effects without the adverse effects commonly associated with psychotropic drugs [93]. The emotional stress that often appears in dental patients can also be altered by using EOs [52,94]. Using aromatherapy of *Lavender oil* in the waiting area or *Citrus oil* to reduce salivary cortisol and pulse rate has also been helpful in stress management. A study shows that using a candle warmer diluted with *Lavender oil* in dental offices before procedures increased sedation, decreased stress and anxiety, and improved overall mood [95]; it was found to be useful in third molar extractions and orthognathic surgeries because of its anxiolytic properties [21,88,96,97]. A study by Sioh Kim et al. showed that *Lavender oil* also reduces injection pain [98].

#### 2.6.3. Wound Dressing

EO-infused wound dressings are a type of wound care product that incorporates EOs such as *Tea tree oil*, *Lavender oil*, and *Eucalyptus oil* into the dressing material. They are believed to have antimicrobial and anti-inflammatory properties that can aid healing and reduce the risk of infection. Budzynska et al. found wound dressings containing EOs that can provide better therapeutic effects. Furthermore, these effects were more substantial when the dressings were stored at 4 degrees Celsius for seven days. As a result, EOs can provide healing following oral surgical procedures [99]. Wound dressing with EOs, the possession of antibiofilm activity during dental implants, and the possibility of being used instead of methylparaben in allergy cases are found to be effective, but further clinical trials are necessary to rule out side effects [85]. Another study by Gheorghita et al. shows that the obtained samples containing *Fennel*, *Peppermint*, *Pine*, and *Thyme oil* have good antimicrobial properties against *S. aureus*, *E. faecalis*, *E. coli*, *P. aeruginosa*, and *C. albicans* [100]. In treating burned wounds, EOs extracted from eucalypt, ginger, and cumin, prepared as hydrogels, have shown high antibacterial activity, superior water retention, mild swelling, and a significant effect on skin repair [101].

#### 2.6.4. Dental Implants and Periodontics

It has been shown that EOs significantly inhibited the adherence of *C. albicans* on dental implants and low results on cover screws [102,103] and are also helping people using polymerized polymethyl methacrylate dental devices [54]. The frequency of drug-resistant strains and new pathogens rises daily, and EOs have shown an excellent antifungal alternative [104]. *Eucalyptus oil* has shown plaque reduction activities and antibacterial effects against *P. gingivalis* and *S. mutans*, which cause periodontitis and other oral pathologies [105]. Herbs, as well as *Coconut oil* [106], are helpful in the treatment of soft tissue and in treating periodontitis and gingivitis because of their biological and medicinal properties, low costs, and high safety margin [107,108]. Plant extracts also inhibit dental plaque growth, lowering biofilm adhesion and reducing oral disease symptoms [109,110]. A study by Mostafa et al. showed great gingival and periodontal status improvement after using a derma pen treated with *Sesame oil* and *Coconut oil*; also, the alkalis present in saliva can react with the oil, causing saponification which reduces the adhesion of plaque and inflammation [111].

#### 2.6.5. Odontology and Prosthodontics

A glass EO-based ionomer cement has been shown to have potent antimicrobial properties by inhibiting both *S. mutans* and *C. albicans* [112]. Plant extracts, EOs, and phytochemicals have also been studied to have the ability to prevent bacterial adhesion [44] so that soft tissues can maintain their state of health. Another experimental product containing *Zataria multiflora* EO effectively reduced the fungal load and the local inflammation. Patients with prosthetic stomatitis also healed entirely or partially after using the EO-containing gel [72,78]. Additionally, they are as effective as CHX at controlling gingiva inflammation after six months of use [113].

#### 2.6.6. Endodontics

Removing microorganisms from the root canal system is crucial for successful endodontic treatments. If not eliminated effectively, microorganisms can lead to resistant infections and poor healing. *E. faecalis* is commonly found in root canals diagnosed with apical periodontitis and is a primary pathogen in secondary endodontic infections. It can survive in harsh, nutrient-deficient environments and grow as a biofilm on root canal walls. The instrumentation and irrigation of the root canals have shown success in canal disinfection. A study by Gokalp et al. showed that a material combined with calcium hydroxide and two EOs (*M. spicata* and *O. dubium*) had significant antimicrobial activity [114]. Another study by Marinkovic et al. used a product containing *C. martinii* and *T. zygis*, which showed antimicrobial activity in the root canals of extracted teeth [115]. Regarding the permanent filling of the root canals, new resin sealers containing natural oils show potential in endodontics due to their favorable physical and chemical properties, antimicrobial effects, and compatibility with cells compared to a commonly used commercial sealer [116,117]. New studies are showing that nano-emulsions EOs-based are showing promising activity against microorganisms for root canal and periodontal treatments.

#### 2.6.7. Preservatives

EOs were more effective in inhibiting certain microorganism strains than extracts and methylparaben. Therefore, they could be used as a substitute for methylparaben in cosmetic emulsions and as a preservative in dental products for patients allergic to methylparaben. However, more clinical trials are needed to determine the safety and efficacy of using EOs as a preservative in products injected into the human body, as studies are not sufficient yet [108].

Other therapeutic effects of the most commonly used EOs are shown in Table 4.

### 2.7. Toxicity

Marketable EOs may result in toxicity due to factors such as improper product management, specific ingredients, overuse, improper use, the potential for sensitization or anaphylaxis, and lack of scientific evidence. Therefore, it is crucial to be aware of the potential adverse effects in addition to the intended use. Some studies have reported additional side effects, such as skin irritation and allergic reactions when using EOs. They are seen as “harmless” because of their natural provenance [134], but often, they can lead to several toxic effects, as seen in Table 5.

The maternal reproductive toxicity of some EOs has also been a significant concern, and using these during pregnancy is highly controversial. Pregnant women often choose to use herbs, herbal preparations, or oils instead of conventional medication to alleviate symptoms associated with pregnancy (such as morning sickness, nausea, vomiting, and heartburn) due to concerns about the potential adverse effects on the unborn child. Nevertheless, some constituents, such as methyl eugenol, cinnamaldehyde, camphor, and thujone, cause maternal toxicity, teratogenicity, embryo-fetotoxicity, or anti-angiogenic effects [135].

A study in Iran used aromatherapy techniques such as inhaling, massaging, foot baths, birthing pools, acupressure, and compresses on women in labor. *Lavender oil* was the most commonly used EO in the study, alone or in combination with other oils. The majority of studies included found that aromatherapy had a positive effect on reducing pain and anxiety during labor [136].

## 3. Conclusions

This review focuses on the most recent information on the effects of EOs on the oral microbiome. Within the scope of this paper, EOs have the potential to be used as therapeutic agents for many oral diseases due to their antimicrobial, antibacterial, antiviral, antifungal, and anti-inflammatory properties. Although these activities are well established, their natural effect is weaker compared to antibiotics; therefore several EO combinations can be implemented to achieve microbial stabilization.

Due to a lack of clinical evidence to support the efficacy of EOs, they are currently only used as alternative therapies. Therefore, further research on the clinical use of EOs in treating oral pathologies is needed.

## Figures and Tables

**Table 1:** Extraction methods of EOs and their meaning.

Method	Description	Reference
Supercritical fluids extraction	A supercritical fluid is a substance maintained above its maximum pressure and temperature, and by adjusting these two, it is possible to manipulate the fluid’s viscosity and density.	[29]
Subcritical fluids extraction	It has lower temperatures and pressure, environmental compatibility, shorter extraction time, and good selectivity.	[30]
Hydrodistillation	Plants are placed in a distiller mixed with water; by heating it, the oil will vaporize with the water vapors.	[26]
Steam distillation	There is a steam generator that passes through the plant before condensation.	[11,31]
Hydrodiffusion	The plants are soaked in the solvent before extraction, and the solvent is evaporated afterward.	[32]
Solvent extraction	Produces an oil extract by having different vapor pressures.	[33,34]
Solvent-free microwave extraction	Microwaves are used to heat the sample’s surface and to promote structural changes.	[35]

**Table 2:** EOs’ chemical compounds and their bacterial target.

EOs	Compounds with Antimicrobial Effect	Inhibited Microorganism	Reference
Thyme oil	Thymol P-cymene Linalool	*S. aureus*	[47,48]
Clove oil	Eugenol Eugenol acetate Caryophylene	*C. albicans*	[21,49,50]
Lavender oil	Linalool Terpineol Caryophyllene Limonene Pinene	*S. aureus* *C. albicans* *E. coli*	[28,51,52]
Cinnamon oil	Cinnamaldehyde Eugenol Linalool	*S. aureus* *S. sobrinus* *S. mutans* *L. acidophilus* *C. albicans* *P. gingivalis* *E. coli*	[53,54]
Eucalyptus oil	Pinene Limonene Terpineol	*S. aureus* *S. mutans*	[55,56]
Lemon oil	Pinene Caryophyllene Linalool Citral Terpineol Limonene	*C. albicans* *S. aureus* *E. coli*	[55,57,58]

**Table 4:** Most commonly used EOs in dentistry.

EOs	Therapeutic Effect	Reference
Clove oil	antibacterial antiseptic antiviral improves halitosis prevents periodontitis reduces dental pain	[41,50,56]
Lavender oil	antibacterial antiseptic anxiolytic reduces dental pain	[8,15,28]
Cinnamon oil	anti-inflammatory antifungal antiseptic	[54,118,119]
Eucalyptus oil	anti-carcinogenic antibacterial antiviral cytotoxic	[15,37,56,120]
Tea tree oil	alleviates bleeding gums antibacterial decreases tooth decay	[37,121]
Ylang-ylang oil	anti-inflammatory antibacterial antianxiety	[37,122,123]
Lemon oil	antibacterial antifungal decreases tooth decay promotes tissue growth reduces halitosis	[37,38,57,58]
Coconut oil	antimicrobial reduces plaque adherence	[37,106]
Spearmint oil	improves halitosis soothes mouth tissues	[56,124]
Curcuma oil	anti-inflammatory antimicrobial antiviral	[89,125,126]
Citrus oil	antianxiety antimicrobial decreases tooth decay reduces plaque adherence	[96,124,127]
EOs from propolis residues	antibacterial antimicrobial antioxidant	[128,129]
Thyme oil	antifungal antiviral (HSV1 virus) bacteriostatic	[28,47,130,131]
Sesame oil	antifungal antimicrobial antiviral reduces plaque adherence	[106,111]
Rosemary oil	anti-inflammatory antitumor antiviral bacteriostatic	[23,132,133]
Peppermint oil	antibacterial antimicrobial antiviral reduces plaque adherence	[56,125,134]

**Table 5:** Toxic effects and doses of several compounds found in EOs.

Toxic Compound	Effect	Toxic Dose	Reference
Pulegone	hepatotoxic irritant carcinogenic	>460 mg/bw/day	[9,24]
Methyl eugenol	carcinogenic genotoxic	>37 mg/kg bw/day	[9,67]
Eugenol	genotoxic allergic contact dermatitis asthma rhinitis	>35 mg/kg bw	[8,9,67]
Camphor	gastrointestinal disorders neurotoxic seizures	>30 mg/kg bw	[9]
Thujone	neurotoxic	>25 mg/kg bw	[9,58]
Limonene	irritant carcinogenic nephrotoxic	>500 mg/kg bw/day	[38,50,58,90]
Linalool	ataxia narcosis	>2.79 g/kg/day	[58]
Terpinene	mutagenic	>3.65 g/kg/day	[58]
Pinene	irritant	>5 g/kg/day	[58]
